# Investigation of Anterior Teeth Fractures Among Students Aged Between 8 and 13 Years in Hyderabad, India

**DOI:** 10.7759/cureus.53131

**Published:** 2024-01-28

**Authors:** Pavani Bellamkonda, Anitha Akkaloori, Ramesh Kumar Koothati, Trinita H, Mansi Sharma, Vyshakh Krishna

**Affiliations:** 1 Public Health Dentistry, Sathyabama Dental College and Hospital, Chennai, IND; 2 Public Health Dentistry, Government Dental College and Hospital, Hyderabad, IND; 3 Oral Medicine and Radiology, Government Dental College and Hospital, Vijayawada, IND; 4 Dentistry, Sathyabama Dental College and Hospital, Chennai, IND

**Keywords:** school children, tooth fracture, anterior teeth, dental injury, dental trauma

## Abstract

Introduction

Trauma involving anterior teeth stands as a prevalent type of dental injury among school-age children, impacting physical, psychological, and social well-being. This study aimed to assess the occurrence of fractures in anterior teeth among school children in Hyderabad and its associated risk factors.

Materials and methods

This research incorporated a cross-sectional analysis, involving 2046 children in the age group of 8 to 13 years from different schools in Hyderabad City. Alongside clinical evaluations, all participants completed a questionnaire regarding traumatic dental injuries.

Results

Results indicated a prevalence rate of 8.5%, notably higher among younger boys. Factors such as lip competence coverage, increased overjet, and malocclusion with maxillary incisor proclination were associated with a heightened risk of such injuries. The peak incidence was observed at age 12, with fractures involving enamel and dentin being the most common type, predominantly affecting the maxillary central incisors.

Conclusion

The findings emphasize the significance of educational programs aimed at enhancing awareness and understanding of dental injuries among parents, students, and school staff.

## Introduction

Besides dental caries, dental injuries are the prevailing dental issue among school children, occurring in both their primary and permanent teeth. Trauma stands out as a significant cause of these injuries. These injuries not only impact the teeth but also affect the supportive structures like the maxilla, mandible, and other facial elements. Furthermore, dental trauma can profoundly influence an individual's quality of life, directly or indirectly impacting facial appearance, mastication, speech, and tooth alignment. This impact extends to the person's physical, psychological, and social well-being [1].

Participation in outdoor games and sports at school significantly contributes to the physical and psychological growth of children. However, when these activities lack supervision or involve aggressive behavior, falls, or improper movements, the risk of injuries increases significantly. Dental injuries, particularly to primary teeth, can lead to abnormalities in the development of permanent teeth. Studies indicate that a substantial percentage, ranging from 25% to 69%, of permanent tooth abnormalities stem from traumatic injuries to primary teeth [2].

Traumatic injuries of the teeth can result in lesions involving pulp and periodontal tissues. Several studies have corroborated this, indicating that a notable percentage (up to 34%) of dental injuries sustained by adults originated during their childhood and adolescence [3]. The impact of a fractured anterior tooth is significant, not only for the child but also for their parents, emphasizing the emotional and practical challenges associated with such an occurrence [4].

Andreason et al. have highlighted a significant rise in the occurrence of traumatic dental injuries over the past two decades. They predict that this increase will likely lead to higher incidence rates compared to common dental issues like dental caries and periodontitis in the future [1].

Encouraging education for teachers and caregivers, who play a crucial role in the immediate response to children's traumas, is highly advisable. Educational programs that highlight the importance of immediate treatment and precautions for the prevention of dental injuries should be designed. To enhance their effectiveness, these programs would benefit from scientific data that showcase the risk factors associated with dental injuries involving anterior teeth [5,6]. With this aim, the current research aimed to assess the prevalence of traumatic injuries in anterior teeth among school-age children in Hyderabad City and establish correlations with predisposing risk factors.

## Materials and methods

As part of a school dental screening program, this cross-sectional study aimed to gather baseline data on the prevalence of dental injuries among school children. The study included 2046 school children, aged between 8 and 13 years, encompassing both genders and students from both primary and high schools within Hyderabad City.

We utilized a multistage sampling method to select the study subjects. Initially, we acquired a city map and divided it into five zones. Subsequently, we compiled a list of schools in each zone, randomly selecting one school from each. To ensure that the sample accurately represented the school population in terms of size and distribution across zones, a random sampling method was employed.

In the subsequent stage, at least 400 children were selected from the previously chosen schools. Prior to conducting the study, permission was obtained from the school authorities. Inclusion criteria involved children aged between 8 and 13 years, with fully erupted anterior teeth. Exclusions encompassed children undergoing current or recent orthodontic treatment and those with partially erupted or missing anterior teeth due to reasons other than trauma. 

A structured questionnaire was used to collect specific information, including the age at the time of injury, the nature of the injury, incisal overjet, molar relation, and lip closure. The measurement of overjet was taken with a CPITN probe [7]. Additionally, socio-demographic details were recorded.

**Figure 1:**
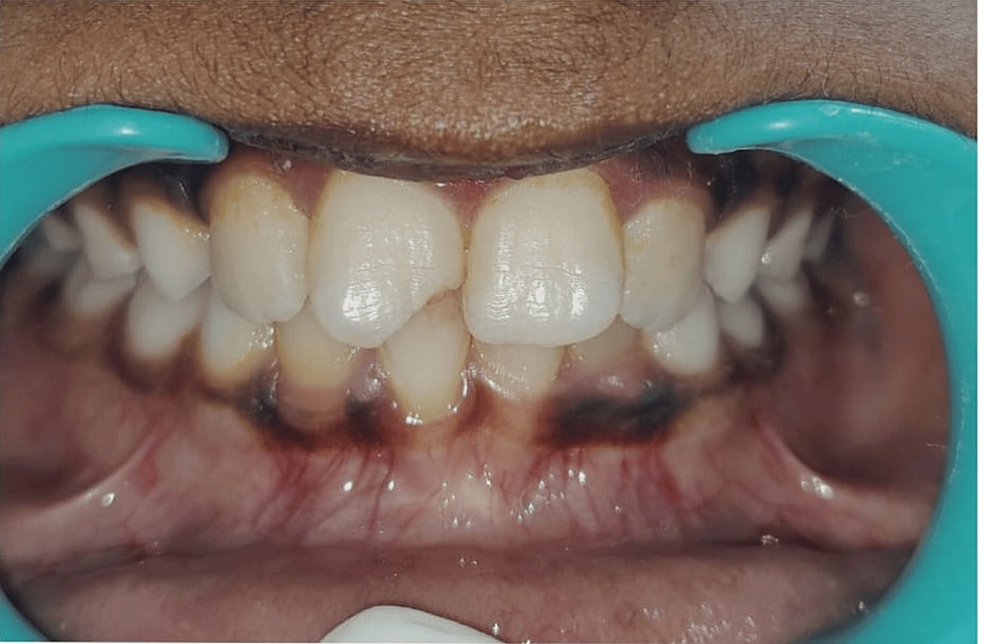
Picture showing fractured permanent right central incisor involving enamel and dentin

**Figure 2:**
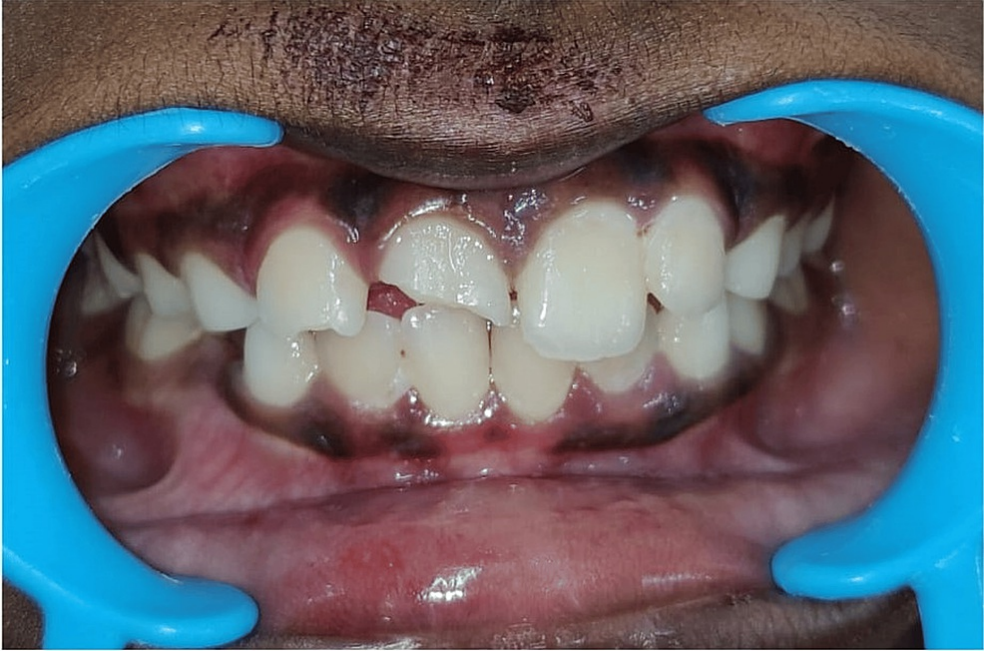
Picture showing fractured permanent right central and lateral incisors involving enamel and dentin

In this study, Andreasen's Classification of Anterior Teeth fractures [1] was employed to assess dental injuries. Root fractures were excluded from the analysis due to the absence of radiographs (Figures 1, 2). The statistical analysis of the collected data was conducted using IBM SPSS Statistics for Windows, Version 13 (Released 2005; IBM Corp., Armonk, New York, United States). The qualitative data were compared using the chi-square test to ascertain statistical significance, with a predetermined significance level set at P < 0.05.

## Results

The total number of children examined was 2046, among whom 174 presented with fractures in their anterior teeth. The prevalence of dental injuries in our study was found to be 8.5% (Table 1). Notably, these injuries were more prevalent between 8 and 12 years, with the highest frequency observed at 12 years, constituting 28.7% of the cases (Table 2).

**Table 1:** Prevalence of dental trauma among study subjects *Statistically significant

Gender	Number of Children examined	Prevalence of anterior tooth fracture n (%)
Male	1075	107 (9.9)
Female	971	67 (6.9)
Total	2046	174 (8.5)
P=0.016^*^		

**Table 2:** Prevalence of dental traumatic injuries in relation to the age of the study subjects NS: Not significant

Age in years (n)	Male (n=1075)	Female (n=971)	Prevalence
8	6 (5.6%)	4 (6.0%)	10 (5.7%)
9	23 (21.5%)	15 (22.4%)	38 (21.8%)
10	26 (24.3)	17 (25.4%)	43 (24.7%)
11	17 (15.9%)	12 (17.9%)	29(16.7%)
12	31 (29.0%)	19 (28.3%)	50 (28.7%)
13	4 (3.7%)	0 (0%)	4 (2.4%)
Total	107 (100%)	67 (100%)	174 (100%)
P= 0.48^NS^			

The incidence of injuries was notably lower (39.7%) when lip competency was adequate compared to cases with inadequate lip coverage (60.3%). Among different occlusions, Class II Div 1 exhibited the highest number of dental injuries (14.07%), whereas Class II Div 2 presented the lowest incidence (2.6%). Additionally, dental injuries were more prevalent among children with an overjet greater than 5.5 mm, with a prevalence rate of 28.62%.

**Figure 3:**
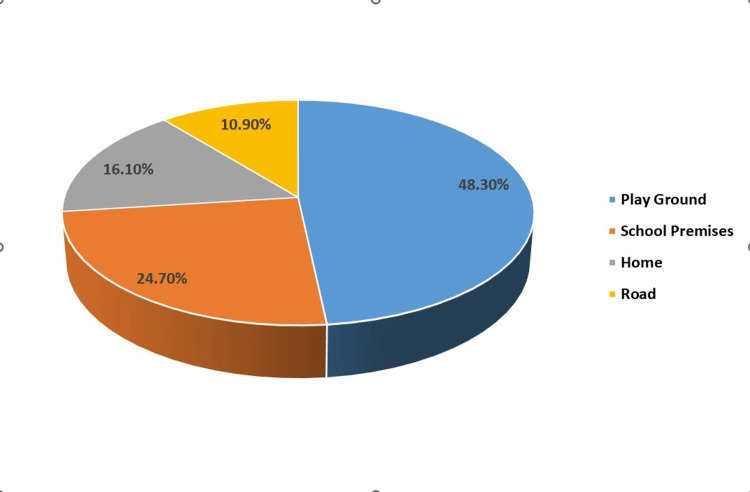
Place/Location of occurrence of dental trauma

**Figure 4:**
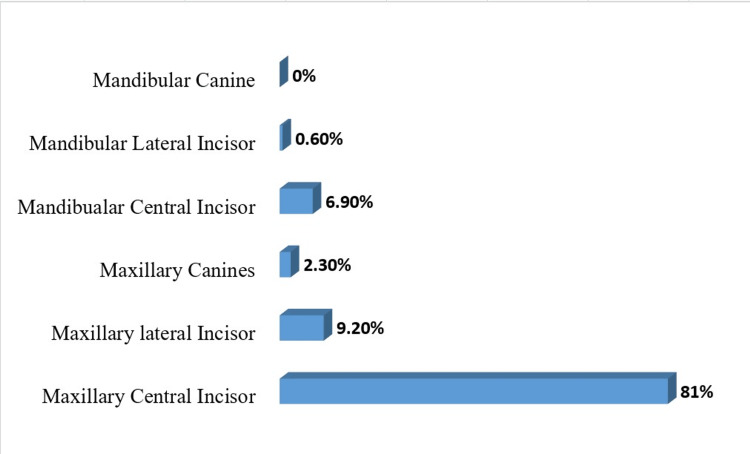
Type of teeth involved in traumatic dental injuries

In terms of the location where injuries occurred, the majority (48.3%) took place at playgrounds, followed by incidents at school (24.7%) and home (16.1%) (Figure 3). Among the types of dental injuries observed, the most common was a fracture involving only the enamel and dentin (41.9%), followed by fractures involving only enamel (24.7%) (Table 3). Sports-related injuries accounted for 53.86% of cases. Maxillary central incisors were the teeth most frequently affected (81%) (Figure 4).

**Table 3:** Type of traumatic dental injuries with gender of the study subjects *Statistically significant

Type of fracture	Male	Female	Total	P-value
Discoloration of teeth	16 (14.9%)	12 (17.9%)	28 (16.1%)	0.16
Crown fracture involving enamel	31 (29.0%)	12 (17.9 %)	43 (24.7%)	0.024^*^
Crown fracture involving enamel & dentine	44 (41.1%)	29 (43.3%)	73 (41.9%)	0.036^*^
Crown fracture involving enamel, dentine & pulp	12 (11.2%)	9 (13.4%)	21 (12.1%)	0.358
Mobility	4 (3.7%)	5 (7.5%)	9 (5.2%)	0.185
Total	107 (100%)	67 (100%)	174 (100%)	0.046

## Discussion

The primary objective of this study was to evaluate the prevalence of traumatic dental injuries among children aged 8 to 13 years residing in Hyderabad city. This specific age range was chosen due to the increased involvement of children in outdoor activities during this period. Additionally, it aligns with a crucial phase of physiological development in children, making it a significant time frame to observe and assess the prevalence of such injuries.

In our research, the observed prevalence of anterior teeth fractures stood at 8.5%. Comparable studies showed a range of prevalence rates, spanning from 4.1% documented by Nik-Hussein et al. [8] to 23.3% reported in the study conducted by Soriano et al. [9]. Some investigations indicated higher prevalence rates, ranging from 13.8% to 20.9% [10-13], while others, like those by Hedge (7.3%) and Patel (8.79%), reported lower rates [14,15]. These variations in prevalence rates among studies might arise from differences in the age groups of the study subjects, sample sizes, classifications used for defining dental injuries, and differences in the types of sports commonly played.

In our study, the prevalence of anterior tooth fractures in males compared to females was 1.59:1, aligning with findings from other studies that also noted a higher occurrence among males [14,15].

Similarly, our research revealed that the highest incidence of dental injuries was at 12 years in both genders. This finding is consistent with previous studies, which indicated that most fractures to the anterior teeth occurred in children between 8 and 14 years [14-16].

We observed a higher prevalence of anterior tooth fractures in children with incompetent lips (60.3%). Previous studies reported that inadequate soft tissue/lip coverage was associated with an increase in the frequency and severity of incisor fractures (24.19% and 38%) [15,17]. However, a study by Traebert et al. did not find a significant association between lip competence and anterior tooth fractures [18]. From these findings, it can be understood that correction of malocclusion and improving the lip closure might reduce the risk of anterior tooth fractures.

Additionally, tooth fractures were most frequently observed (14.07%) in children with maxillary incisor proclination (Angle’s class II Division I malocclusion), which reflects findings from earlier studies conducted by different researchers [14,15].

The relation between increased incisal overjet and anterior tooth fractures has been widely investigated by various researchers, yielding conflicting findings. In this study, a statistically significant association was identified between overjet and the frequency of dental injuries, particularly noting a higher occurrence among children with an overjet exceeding 5.5mm (28.62%). This aligns with the observations made by Baldava (32%) and Patel MC (22.22%), who similarly noted that individuals with increased overjet were more susceptible to dental injuries compared to those with normal overjet [12,15]. However, studies conducted by Hegde et al. did not establish any correlation between different overjet sizes and the frequency of dental injuries [14].

In our research, the majority of dental traumatic injuries (53.86%) were attributed to "sports,", contrary to “fall” being a major cause (35.7% to 61.8%) in other similar studies [11,13-15,19]. Notably, the usage of protective equipment in schools during contact sports plays a major role in preventing such injuries. Moreover, a considerable percentage of school children couldn't recall the cause of trauma.

In our investigation, the most observed form of dental injury was identified as fractures affecting a single tooth involving enamel and dentine (41.9%). The finding differs from those found in earlier studies conducted by Patel (46.7%), Bhayya (55.6%), Vanka (Nonvital tooth 50.7%), and Ravishankar (74.1%), where enamel fractures and non-vital tooth were the most common type [15, 20-22]. However, it is important to note that in certain studies, the most prevalent type of injury reported involved fractures affecting both the enamel and dentin [14,23-25].

Our present research identified the playground as the primary site for dental injuries (48.3%), closely followed by incidents occurring on school premises. This finding corresponds with earlier studies [18, 20, 26-28]. However, Gupta, Patel, and Bastone reported a different trend, noting that most injuries happened at home (43.8% to 60%). These discrepancies in findings among studies imply variations in the settings or contexts where these incidents occurred [11,15,29].

In our research, the maxillary central incisors were frequently affected (81%), followed by the maxillary lateral incisors. This aligns with observations made in earlier studies by Nik-Hussein (78.0%) and Ravishankar (93.8%) [8,22]. The increased vulnerability of the maxillary central incisors could be attributed to their early eruption, keeping them at risk for a longer duration compared to the lateral incisors. Additionally, the impact on mandibular teeth tends to be more dispersed due to the flexible connection of the mandible to the cranial base, rendering mandibular incisors relatively less susceptible to injury than maxillary incisors.

Limitation(s)

In our study, the diagnosis of dental trauma relied solely on clinical examination, without incorporating radiographic imaging. This approach may have led to the omission of certain fractures, such as root fractures, which could not be identified without radiographs.

## Conclusions

Traumatic dental injuries have wide-ranging impacts beyond mere structural damage. They significantly influence various aspects of life, including aesthetics, chewing abilities, and speech. The findings from our study highlight that children in the mixed dentition phase are particularly susceptible to these injuries.

This underscores the critical need for educating first responders about managing traumatic dental injuries. Creating awareness about proper techniques for cleaning and transporting the affected tooth can be instrumental in preserving its viability. This, in turn, enhances the chances for dental professionals to successfully replant the tooth at the earliest opportunity, ultimately improving the outcome for the affected individual.
